# When Sensor-Cloud Meets Mobile Edge Computing

**DOI:** 10.3390/s19235324

**Published:** 2019-12-03

**Authors:** Tian Wang, Yucheng Lu, Zhihan Cao, Lei Shu, Xi Zheng, Anfeng Liu, Mande Xie

**Affiliations:** 1 College of Computer Science and Technology, Huaqiao University, Xiamen 361021, Chinamail_luyu@163.com (Y.L.); mail_caozhihan@163.com (Z.C.); 2 Key Laboratory of Computer Vision and Machine Learning (Huaqiao University), Fujian Province University, Xiamen 361021, China; 3 College of Engineering, Nanjing Agricultural University, Nanjing 210095, China; lei.shu@njau.edu.cn; 4 Department of Computing, Macquarie University, Macquarie Park, NSW 2109, Australia; james.zheng@mq.edu.au; 5 College of Computer Science and Engineering, Central South University, Changsha 410083, China; afengliu@mail.csu.edu.cn; 6 School of Computer Science and Information Engineering, Zhejiang Gongshang University, Hangzhou 310018, China

**Keywords:** sensor-clouds, WSNs, cloud computing, mobile edge computing

## Abstract

Sensor-clouds are a combination of wireless sensor networks (WSNs) and cloud computing. The emergence of sensor-clouds has greatly enhanced the computing power and storage capacity of traditional WSNs via exploiting the advantages of cloud computing in resource utilization. However, there are still many problems to be solved in sensor-clouds, such as the limitations of WSNs in terms of communication and energy, the high latency, and the security and privacy issues due to applying a cloud platform as the data processing and control center. In recent years, mobile edge computing has received increasing attention from industry and academia. The core of mobile edge computing is to migrate some or all of the computing tasks of the original cloud computing center to the vicinity of the data source, which gives mobile edge computing great potential in solving the shortcomings of sensor-clouds. In this paper, the latest research status of sensor-clouds is briefly analyzed and the characteristics of the existing sensor-clouds are summarized. After that we discuss the issues of sensor-clouds and propose some applications, especially a trust evaluation mechanism and trustworthy data collection which use mobile edge computing to solve the problems in sensor-clouds. Finally, we discuss research challenges and future research directions in leveraging mobile edge computing for sensor-clouds.

## 1. Introduction

With the development of many smart applications in various areas including healthcare, traffic, manufacturing, and farming, sensor-clouds, which connect sensor networks and clouds, are receiving a lot of attention from both the academic and industrial communities. Sensor-cloud removes the need for various applications to use separate sensors, thereby improving the utilization of sensor nodes and providing customized services for users. Based on sensor-clouds, individuals and citizens can pay more attention to leveraging the intelligent sensor infrastructure deployed by providers.

However, sensor-clouds also have some obvious problems, such as communication problems between sensor networks and clouds, additional space, high latency due to a cloud server being too far away from underlying sensor networks [1]. In particular, some aspects, such as how to deal with the third-party trustworthiness problem, data collection being limited by the communication capacity of wireless sensor networks (WSNs), and the coupling security problem, are waiting to be explored. Therefore, it is necessary to explore a new approach and model to solve these problems. We note that mobile edge computing has many advantages, including higher performance, better efficiency, and stronger security. As an extension of cloud computing, mobile edge computing is an emerging hot technology, which is characterized by getting closer to the underlying network than cloud computing, supporting mobility, and having strong computing capacity. It can be said that mobile edge computing is an intermediate state between cloud and WSNs [2]. Mobile edge computing can be regarded as having micro-clouds that move in the middle range of sensor-cloud architecture to achieve safe and reliable data processing and storage.

At present, most of the surveys on sensor-cloud research were written five years ago. There is hardly any sensor-cloud survey based on mobile edge computing. In [3], Atif Alamri et al. only introduced the traditional sensor-cloud architecture, and emphasized the benefits brought by the introduction of cloud computing to WSNs network. Zhu et al. focused on the development and prospects of social sensor-clouds and emphasized the combination of social sensing network and sensor-clouds [4]. Sukanya et al. focused on the data processing framework in sensor-cloud systems [5]. The above papers only reviewed some research directions of sensor-clouds, without comprehensive investigation and analysis, and did not combine a sensor-cloud with the rapidly developing mobile edge computing in recent years. Therefore, for the field of sensor-clouds, it is very necessary to comprehensively investigate and study sensor-clouds based on mobile edge computing.

We surveyed the prospects of introducing mobile edge computing paradigm to a sensor-cloud. We analyzed a number of application scenarios, before we exploited the benefits of introducing mobile edge computing into a sensor-cloud. Specifically, the following three contributions are within this paper.

- The characteristics of sensor-clouds are summarized, and the issues in sensor-clouds are detailed. Compared with other surveys of sensor-clouds, we present the latest research status of sensor clouds based on mobile edge computing.
- We then discuss the edge-related solutions, which aim to address the data management issues (collection, trust, storage etc.) in sensor-clouds.
- The future directions and challenges are highlighted, which justify the need for in-depth research on the mobile edge-based sensor-cloud.

The rest of the paper is structured as follows. The essential system architecture and characteristics for enabled applications in a sensor-cloud are identified, and a descriptive review on data management is presented in Section 2. The advantages of mobile edge computing for sensor-clouds are discussed in Section 3. The key challenges and solutions based on mobile edge computing are introduced in Section 4, and some applications of mobile edge-based sensor-cloud are proposed. Then, we discuss the combination of mobile edge computing and sensor-cloud in data collection in Section 5. Furthermore, some expected solutions and further research directions are highlighted. We then survey our review for mobile edge-based sensor-cloud as a conclusion in Section 6.

## 2. Sensor-Cloud Overview

In this section, we will briefly introduce the definition of a sensor-cloud, and focus on the design ideas and characteristics of the existing sensor-cloud platforms. Then, some related work on sensor-clouds will be described, which raises the possibility of combining a sensor-cloud and mobile edge computing.

### 2.1. Sensor-Cloud Design and Characteristics

A sensor-cloud is the combination of WSNs and cloud computing and controls sensor networks through a cloud for information collection, processing, and storage. Thus, a sensor-cloud combines the advantages of cloud computing and expands the computing capacity, storage capacity, communication capacity, and scalability of traditional WSNs. The underlying sensor network is mainly responsible for collecting data, and complex operations such as data analysis and processing can be delivered to cloud, which reduces the burden on sensor networks and speeds up the data processing rate. In the sensor-cloud enabled applications, users only need to send application requests to sensor-cloud, which automatically distributes and dispatches some sensor networks to provide services in real-time [6].

**Figure 1:**
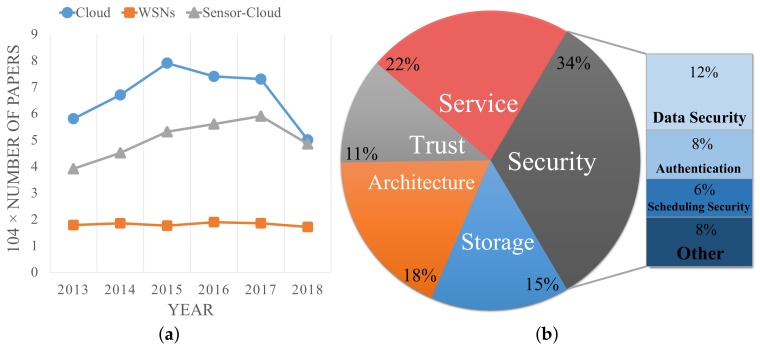
Trends of sensor-cloud research. (**a**) Related work; (**b**) Research directions.

In recent years, a number of research efforts on cloud, WSNs and sensor-cloud have done. Figure 1a shows the tendency of the number of related papers in Google Scholar. Obviously, the studies on cloud are decreasing and the studies on WSNs are relatively stable. However, the trend in sensor-cloud researches was initially on the rise, and has declined over the past two years. According to historical experience, when a mature technology reaches its peak and begins to decline, it soon after enters the stage of a mature application, such as IoT technology. For sensor-clouds, this downward trend is a sign that it is maturing, and this is the perfect time to study its applications and open issues. Figure 1b shows the number of papers in different research directions in sensor-clouds over the past five years in the Science Citation Index and Google Scholar, mainly in service, security, architecture, trust, and storage.

In the light of its characteristics and advantages, the sensor-cloud has become an important field. A model combining WSNs with cloud computing paradigm was proposed in [7]. Ihsan et al. surveyed recent advances in sensor-cloud with data collection [8]. In [9], in order to assess the trustworthiness of both mobile sink nodes and sensors, Wang et al. defined three types of trust, including direct trust, indirect trust and functional trust for sensor-cloud enabled applications, and proposed a Comprehensive Trustworthy Data Collection (CTDC) method. Simultaneously, Liu et al. proposed a game theory method to realize an energy-saving synergetic defense programme for sensor-cloud environments [10]. In [11], a Multi-Method Data Delivery (MMDD) scheme for sensor-cloud users is proposed. In addition, Zhu et al. also combined sensor-cloud with smart cities [12] and social networks [4].

**Figure 2:**
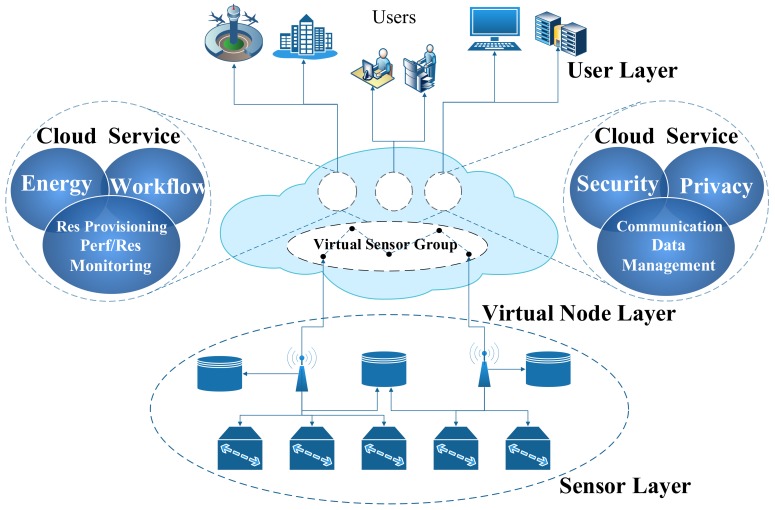
Sensor-cloud architecture.

Figure 2 shows an example of sensor-cloud. It includes the physical node layer, virtual node layer, and user layer from bottom to top. At physical node layer, each sensor node has its own control and data collection mechanism, and sensor nodes in different applications have different functions [13]. For example, sensor nodes in forest monitoring focus on monitoring the necessary information such as temperature and humidity; and sensor nodes in the target tracking require real-time return of information—the target’s geographical location, motion trajectory, etc. This layer is mainly responsible for collecting data and at odds with the upper layer cloud. The virtual node layer is composed of virtual nodes and a cloud. It is mainly responsible for the processing of cloud resources and the scheduling management of physical nodes. When using some sensor-based cloud services, the end user does not need to worry about the specific location of the sensor node due to the existence of the virtual node [14]. Clouds not only provide storage services for users, but also respond to users in a timely manner when there is an urgent request, and provide corresponding services [15]. The uppermost layer is the user layer. Different users can access the resources in a sensor-cloud. Since the resources are shared in a cloud, if the corresponding permissions are obtained, the user can also access other user resources. The user layer provides users with some remote demand services and is compatible with different platform systems [16]. For example, some users request services from a sensor-cloud, which may come from different networks (3G, 4G, 5G, WiFi), different terminals (mobile phones, tablets, computers), or different operating systems (Windows, Linux, Mac) [17].

**Figure 3:**
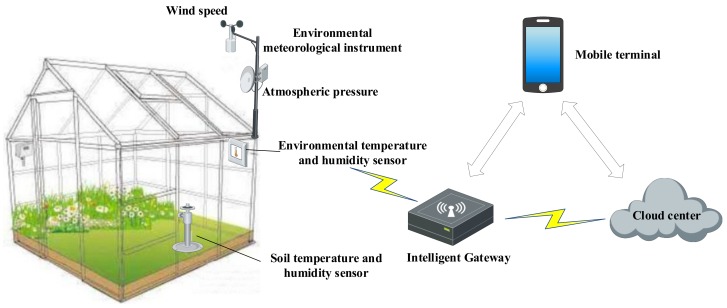
An application of cloud–edge collaboration in a smart greenhouse.

Intelligent agriculture is also a typical case of sensor-cloud. There are three major shortcomings in the development of traditional agricultural informatization, including inaccurate information collected, incomplete network coverage, and uncontrollable production risk. As an advanced stage of agricultural production, intelligent agriculture integrates cloud computing, mobile edge computing, WSNs, and big data technology. It realizes intelligent perception, intelligent pre-warning, intelligent decision-making, and intelligent analysis of an agricultural production environment by relying on various sensor nodes deployed in an agricultural production site, so as to provide accurate planting and visual management for said agricultural production. For example, Figure 3 shows an application of cloud-edge collaboration in the smart greenhouse. As a typical application scenario: the relevant information of the smart greenhouse is obtained and transmitted to cloud, and the model trained by cloud is deployed to the smart gateway after the data are analyzed via cloud. According to the model trained by the cloud and the information collected by sensor nodes, the intelligent gateway can control various devices of the greenhouse in real-time, such as the exhaust fan and electric irrigation system. The user can also set the control logic in cloud via logging onto the cloud system through the terminal, and the cloud will transfer the control logic to the edge control device. Then, the edge control device collects real-time data of the greenhouse environment such as temperature and humidity, light intensity, soil moisture, soil temperature, and wind speed through sensor nodes, and makes intelligent control according to the actual situation.

### 2.2. Existing Implementation

At present, research on sensor-clouds has been carried out for many years. In this section, taking the research on service, security, and trust in sensor-cloud as examples, we analyze the characteristics of some existing sensor-cloud enabled applications and point out the issues sensor-clouds are facing.

*(1)**Service**:* A good user experience is an essential condition for users to choose a sensor-cloud platform [18]. It is an urgent problem to meet the quality of service (QoS) requirements of different users for different applications when there are a large number of concurrent user service requests. The study in [11] proposed a multi-method data delivery (MMDD) scheme for sensor-cloud users with four kinds of data delivery, which was composed of delivery between cloud with sensor-cloud users, delivery between WSNs with sensor-cloud users, delivery between sensor-cloud users with sensor-cloud users, and delivery between edge with sensor-cloud users. The MMDD considers the location, the required data, and the service level agreement of sensor-cloud users to create a data delivery solution. Considering the heterogeneity of the nodes, Misra et al. proposed the QoS-aware sensor allocation algorithm (Q-SAA) that takes into account an assortment of parameters that determine QoS [19]. Thereafter, using an auction-based mechanism, they found the optimal solution for the allocation of a subset of available sensors to achieve efficient target tracking [20].

*(2)**Security**:* Securing network security and user privacy is necessary [21]. Strengthening authentication and encryption mechanisms in different scenarios can help improve the security levels of sensor-clouds [22,23]. Li et al. studied a dynamic, multiple-keys certification method based on game theory against attacks in unmanned and rigorous environments for industrial wireless sensor clouds (IWSC) [24]. In [25], Wang et al. proposed an mobile edge-based model for data collection in sensor-cloud, in which the raw data from WSNs was differentially processed by algorithms on edge servers for privacy computing. A small quantity of the core data was stored on edge and local servers while the rest was transmitted to cloud for storage.

*(3)**Trust**:* Since sensor-clouds were put forward, the research of the trust mechanism has been paid much attention. In vehicular ad hoc networks (VANETs) based on sensor-clouds, it is significant to establish trust among vehicles for guaranteeing integrity and reliability of applications [26,27]. Seyed et al. proposed a fuzzy trust model based on experience and credibility to ensure the security of internet of vehicles (IoV), which executes a range of security detection to guarantee the correctness of the information received from authorized vehicles [28]. In [29], an edge sensor-cloud based intelligent trust evaluation scheme is proposed to comprehensively evaluate the trustworthiness of sensor nodes using probabilistic graphical model. The proposed mechanism evaluates the trustworthiness of sensor nodes from data collection and communication behavior. Moreover, the moving path for the edge nodes is scheduled to improve the probability of direct trust evaluation and decrease the moving distance.

## 3. Mobile Edge-Based Sensor-Cloud

With the development of sensor-cloud, a novel data management mechanism for cloud computing emerged, which expanded the market space of cloud computing and vastly enriched the application prospects of WSNs. However, sensor-cloud also presents new challenges while providing extended services, and corresponding solutions are provided to different challenges.

### 3.1. Why Mobile Edge Computing?

Mobile edge computing was first proposed by the European Telecommunications Standard Institute (ETSI) in 2014 [30]. The basic idea of mobile edge computing is to migrate the cloud computing platform from the inside of the mobile core network to the edge of the mobile access network, so as to achieve the flexible utilization of computing and storage resources. Mobile edge computing pushes mobile computing, network control, and data storage to the edge of the network, enabling compute-intensive and latency-critical applications on edge devices with limited resources.

Compared to the cloud computing pattern, mobile edge computing actually is the extension of cloud computing, for mobile edge computing can stretch cloud computing paradigm to the network edge to compensate for the lack of security in data storage and high latency in service delivery in cloud computing. Mobile edge computing has many advantages and characteristics, including significant role of wireless access, good mobility and scalability, lower latency and location awareness, broader geographical distribution and real-time applications, higher security, etc. [31]. Mobile Edge computing can provide advantages for industry, entertainment, personal computing, and other applications with computing and storage capabilities. Through its integrated data collection, computing, and storage services, the mobile edge-based sensor-cloud system has brought much convenience to individual customers and enterprises [32].

### 3.2. Challenges and Solutions

In terms of communication bandwidth, there is a communication bottleneck between sensor networks and clouds. The bandwidth of wireless communication for each sensor network is limited, and new applications combined with cloud computing often produce a large amount of data and cause communication latency. In the framework of mobile edge computing, each edge node can act as a mobile base station. The local computing power that relies on mobile edge computing first converges and compresses the data when the data in the sensor is delivered to mobile edge nodes, which can reduce the amount of data that needs to be uploaded to a certain extent. A mobile multi-input multi-output (MIMO) network structure can be formed via the collaboration of multiple mobile edge nodes [33]. When a mobile base station is blocked due to the large amount of data, it can forward data to other base stations with the light load, maximizing the data transmission volume of the whole network and reducing the data transmission latency.

Due to the limited resources of the underlying sensor nodes, the probability of failure and error in the complex and harsh deployment environment is generally high, which leads to the generation of malicious nodes and causes interference and damage to the authenticity and integrity of the original data. Faults and errors in the sensor network mainly include node errors, event monitoring errors, and data transmission errors [34]. (a) In terms of node errors, the sensor nodes may fail due to fault or energy exhaustion. Under the mobile edge node framework, the mobile nodes can move to fill the gaps in time, ensuring the integrity of the network topology from both connectivity and coverage; (b) In terms of event monitoring errors, mobile edge nodes which can be close to events can be used to cooperate with fixed nodes for event monitoring, and the monitoring data are used for local decision making to improve monitoring accuracy [35]; (c) In terms of data transmission errors, the reason for data transmission errors is the instability of the wireless link, which can utilize mobile edge nodes to help data collection to reduce wireless multi-hop transmission, or utilize mobile edge nodes in the edge node layer to form a small-world network to build a multi-hop communication path with the least forwarding nodes [36,37].

In addition, sensor node management is also a major challenge in sensor-clouds. A cloud server as the underlying WSNs management platform is far away from the sensor network, and cloud computing lacks the direct management of the underlying terminal sensor nodes [38]. The traditional, remote management, relying only on the cloud platform cannot satisfy the demands of users to take direct control of data, and the delay will be caused by network bandwidth, transmission errors, and other factors [39,40]. In a typical sensor-cloud based on mobile edge computing framework, mobile edge nodes have certain computing, storage, and mobility capabilities, and are close to the sensor network layer, which can better directly manage and control sensor nodes in the sensor network. To avoid high latency problems caused by data interaction with cloud, a part of the computing and storage tasks can be accomplished by mobile edge nodes [41]. Meanwhile, the edge layer can also offload most computing tasks to cloud when computing requires more resources [42].

## 4. State of the Art of Mobile Edge-Based Sensor-Clouds

From the above analysis, we can clearly find problems in current applications that support sensor-clouds. Therefore, based on these issues, we analyzed several effective solutions based on mobile edge computing to address security and service problems in sensor-cloud. This section details how these scenarios can improve the performance of some key aspects of applications that support sensor-clouds. Certainly, there are other considerations besides security and service when it comes to a mobile edge-based sensor-cloud. Then, more discoveries that deserve discussion about mobile edge-based sensor-clouds are summarized in the Table 1.

### 4.1. Improvement of Sensor-Cloud Security

Since sensor-clouds have been proposed, security issues have become a constant concern. Security issues exist in traditional WSNs and cloud computing [43]. To solve these problems, many outstanding studies have proposed individual solutions. In this section, our solutions for security in sensor-clouds will be discussed through a series of mobile edge-based studies.

#### 4.1.1. Data Collection Scheme

**Figure 4:**
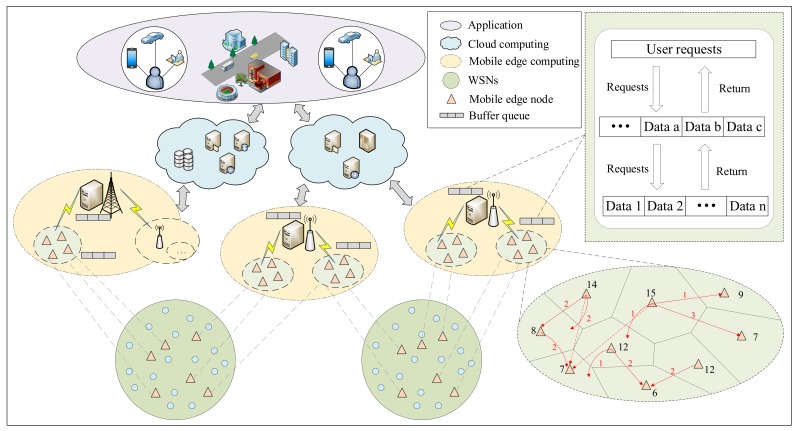
Mobile edge computing-enabled sensor-clouds.

The data collection of a sensor-cloud is limited by the communication capability of WSNs, which can hardly satisfy the real-time data transmission requirements between WSNs and a cloud [44,45,46]. We proposed a new framework based on mobile edge computing where numerous mobile sink nodes serve as the edge layer to eliminate the communication gap between WSNs and a cloud, whose main objective is to achieve the best collaboration among mobile edge nodes and to minimize the transfer delay [47]. In the structure shown in Figure 4, the three layers cooperate with each other to maximize throughput and reduce transmission delay. We use the principle of Voronoi in graph theory to divide the plane into regions and realize the initial setting of the collection structure. Considering the transmission multi-hop and energy problems in the edge layer, we sum each scheduling unit and calculate the average value to plan the path.

The IoT uses embedded systems based on sensors to interact with other systems, providing extensive services and applications for upper-level users [48]. There is no doubt that the data collected by the underlying IoT is the basis of the upper-layer decision and the foundation for all applications, which requires efficient energy protocols [49]. In addition, the data protection and application become an unrealistic target if the data collected is wrong and untrustworthy, further leading to unnecessary energy costs [50,51]. However, traditional methods cannot solve this problem effectively and reliably. To achieve this goal, we designed a novel, energy-efficient, and trustworthy protocol based on mobile edge computing [52]. The mobile data collection path with the maximum utility value is generated via establishing a trust model on edge elements to evaluate sensor nodes, which can avoid visiting unnecessary sensors and collecting untrustworthy data [53]. Simultaneously, in order to ensure data security, we also need to consider the issue of trust in the data collection process, which will be detailed in the next section.

#### 4.1.2. Trust Evaluation Model

For security threats to a sensor-cloud, internal attacks and hidden data attacks account for a large proportion. The trust evaluation model is an effective method to address these attack threats [54,55]. However, there are a lot of problems that need to be concerned, including energy depletion of building trust model in WSNs, finding hidden data attack, and ensuring outer nodes trustworthy, etc. Therefore, a novel trust evaluation model based on mobile edge computing was designed to solve these issues [42]. As shown in Figure 4, one part is a hierarchical trust evaluation model that is used to reduce the resource consumption of trust evaluation model in the sensor network. Another part is the managing and maintaining of entities’ trust relationships based on mobile edge computing. In the experiment, the trust detection period for inner sensors is extended and the trust status analyses in mobile edge computing is an auxiliary means to ensure security of the underlying network. Relative to the periodic detection, our design has some advantages in reducing resource consumption. Though the detection speed of malicious nodes is a little slower than the periodic detection, in-depth data analyses can be done in mobile edge computing that increase the fault tolerance rate and stability of the whole system [56].

Sensor-cloud facilitates data collection, processing, analysis, and storage as a driver of intelligent industrial IoT [57]. However, damaged or malicious sensor nodes can invalidate the collected data and even compromise the normal functioning of an entire IoT system [58]. Hence, it is a critical problem to design an effective mechanism to guarantee the credibility of sensor nodes [59]. However, the existing cloud computing model cannot provide direct and effective management for sensor nodes. Meanwhile, the computation and storage capacity of sensor nodes is insufficient, which makes it difficult for them to execute complex, intelligent algorithms [60]. Therefore, mobile edge nodes with strong computation and storage ability are used to provide intelligent trust evaluation and management for sensor nodes. In [29], an intelligent trust evaluation scheme based on mobile edge computing is proposed to comprehensively evaluate the trustworthiness of sensor nodes via utilizing the probabilistic graphical model. The mechanism proposed evaluates the trustworthiness of sensor nodes from the aspects of data acquisition and communication behavior to effectively ensure the trustworthiness of sensor nodes and decrease the energy consumption [61].

#### 4.1.3. Coupling Security

There are some service conflicts that are collectively called the coupling security problem in sensor-cloud enabled applications, where multiple service requests are contemporaneously received by a physical sensor node. This coupling security problem can lead to service failures and system security threats [73]. As shown in Figure 4, a buffer queue was designed to solve this problem in the edge layer, which can return the result in a cloud layer directly to improve resource usage. Then, the Kuhn–Munkre algorithm is extended to obtain the original allocation of resources. The final step is to confirm whether the initially allocated resources can be rescheduled, which means that resource utilization needs to be further improved to achieve efficient resource scheduling [69].

#### 4.1.4. Storage Security

**Figure 5:**
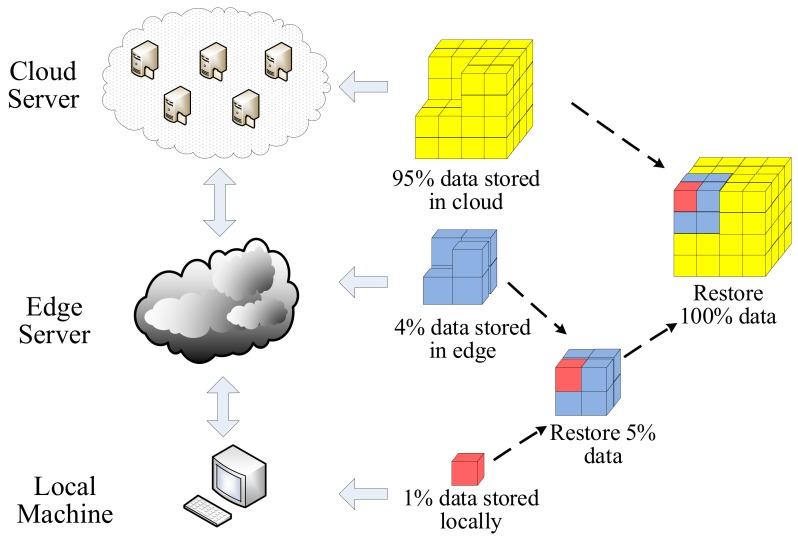
Applying the three-layer storage scheme.

With the rapid growth of all kinds of data, the emergence of cloud storage has aroused the attention toward infrastructure architectures, storage security, and so on. In public cloud applications, cloud service provider owns and manages infrastructure [74,75]. It shows that users’ data cannot be controlled by themselves, and the security of data are at great risk [76]. Users’ data are very likely to be delivered to the parties who are not trusted. In order to protect user privacy, we proposed a three-layer storage (TLS) mobile edge-based scheme. This scheme can ensure that users have data management ability and protect data security to a certain extent. As illustrated in Figure 5, the framework mainly utilizes the data storage and processing capability of edge server. In the three-layer structure, we use Hash–Solomon code to decide to store the minimum scale data locally (for example, 1%). The remaining data is properly separated and uploaded to the edge server (for example, 4%) and the cloud server (for example, 95%), which can protect the privacy and security of users’ data [77,78].

### 4.2. Improvement of Sensor-Cloud QoS

In this section, we focus on the QoS solution for a sensor-cloud. Many applications in sensor-clouds are data-intensive, delay-sensitive, and real-time. QoS (delay, feedback, cost, price, etc.) for delay-sensitive applications must be ensured [79]. For example, in the monitoring of a forest fire, if the delay is too long or the feedback is too slow, it cannot play the role of preventing the fire, thereby causing loss. We can discover problems in time and prevent accidents and save lives by real-time remote monitoring. We utilized mobile edge computing model to solve the following issues.

#### 4.2.1. Dynamic Edge Service

The introduction of mobile edge computing can effectively meet the strict QoS requirement, as it puts computing, storage, and network resources closer to users [80]. Yousefpour et al. proposed a framework called FOGPLAN for dynamic edge service of supporting QoS, which focuses on dynamically deploying application services on the edge layer, or previously published application services deployed on edge nodes to realize low delay and the QoS requirement while minimizing costs [72].

The combination of cyber physical system and cloud computing has attracted great attention from academia and industry, which makes a novel kind of application and service possible. However, due to the relatively long distance between remote cloud and terminal nodes, cloud computing cannot provide valid and immediate management for terminal nodes, leading to security vulnerabilities. In [81], we were the first to propose a novel trust evaluation mechanism based on crowd-sourcing and intelligent mobile edge computing for satisfying QoS. Mobile edge users with relatively strong computing power and storage capacity are exploited to provide direct management for terminal nodes. Mobile edge users can obtain all kinds of information about terminal nodes through close access to terminal nodes, and estimate whether the node is trustworthy. Then, two incentive mechanisms were proposed, namely, the trustworthy incentive mechanism and the QoS-aware trustworthy incentive mechanism, to motivate mobile edge users to conduct a trust evaluation. The goal of the first one is to motivate edge users to upload real information about their capabilities and costs. The second one aims to motivate edge users to make trustworthy efforts to execute tasks and report results [82].

#### 4.2.2. Minimization of Cost and Maximization of Profit

Edge-aided IoT solves the resource constraints of IoT devices in computing, storage, and energy capacity, and enables computation-intensive and delay-sensitive tasks to be offloaded to the mobile edge nodes connected to the IoT gateway [83]. However, due to the high cost of mobile terminal equipment, there is a primary issue that a higher financial budget is required to ensure QoS. At present, some researches focusing on QoS aim to minimize the cost and maximize the profit of cloud service providers and edge service providers.

A truthful pricing policy for crowd-sourcing services provided by mobile edge cloud was proposed in [84], which aimed to minimize the overall cost of devices while ensuring the QoS in the meantime. In [71], Yao et al. addressed the joint optimization problem to minimize the system cost while ensuring QoS requests, transformed as a mixed integer nonlinear programming (MINLP) problem, and designed an approximate algorithm as a solution. They transformed the MINLP problem into a convex optimization problem by relaxing its integer variables, and then designed an integer recovery scheme to obtain the feasible solution.

## 5. Open Research Issues

In previous sections, we summarized the latest research on sensor-cloud and sensor-cloud enabled applications based on mobile edge computing, and analyzed the application advantages of mobile edge computing model in this system. In this section, we discuss node trust evaluation, trustworthy data collection, and a trustworthy data filter based on mobile edge computing in sensor-clouds, and identify future directions.

### 5.1. Mobile Edge Computing for Trust Evaluation

The data comes from nodes, so it is necessary to effectively and comprehensively evaluate the underlying physical nodes to identify faulty and malicious nodes. The traditional method relies too much on the trustworthy cloud center, but the cloud is far away from the sensor network, and the information on the sensor network is not adequately controlled and not timely. It is difficult to fully grasp the state of underlying network, which cannot provide a reliable evaluation of the underlying sensor network. Mobile edge nodes are widely distributed, and the edge node layer is closer to WSNs than cloud, which can comprehensively and timely acquire various state information in the data collection process of the sensor network, thereby objectively evaluating the nodes in the sensor network. Trust evaluation in the sensor network can be divided into two categories: direct trust evaluation and indirect trust evaluation.

**Figure 6:**
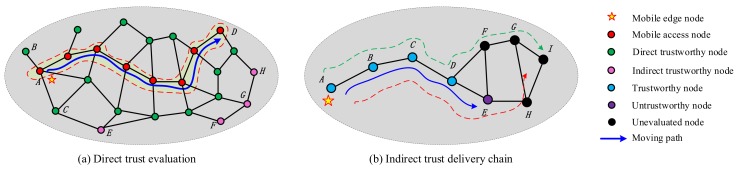
Trust evaluation based on the mobile edge node. (**a**) Direct trust evaluation; (**b**) Indirect trust delivery chain.

Figure 6a shows an example of direct trust evaluation. The mobile node is the near node *A*, and nodes *B* and *C* are direct neighbors of node *A*; then, the mobile node can get direct trust evaluation of nodes *A*, *B*, and *C*. Whereas, for node *D*, since the mobile node and *D* do not interact directly, the direct trust evaluation cannot be obtained under the traditional method, and can only be obtained indirectly through the trust delivery of other intermediate nodes. However, through the movement of the edge node, it is assumed that it moves from node *A* in the figure to the vicinity of node *D*, in which the edge node establishes direct communication relationship with many sensor nodes, so as to conduct direct trust evaluation on them. As shown in the figure, only nodes *E*, *F*, *G*, and *H* cannot be evaluated for direct trust. This is a new method, different from the existing method, and the introduction of mobile edge nodes can greatly increase the chances of the direct trust evaluations of nodes.

For the indirect trust evaluation of nodes, the traditional method is to first find a communication path and then calculate the chain trust delivery on this path. But this type of recommended and transitive type trust calculation is awfully unreliable, and multi-hop trust delivery is an easy way to generate distortion and inaccuracy in a trust evaluation. In the mobile edge node model, the chain of trust delivery can be shortened as much as possible. As shown in Figure 6b, if the mobile edge node is starts from *A*, the trust evaluation chain for the node *I* is *A→B→C→D→E→H→I* or *A→B→C→D→F→G→I*. In the case of moving, assuming that it moves to the node *E* along the blue arrow that happens to be an untrustworthy node, it can roll back a node to build a trust delivery chain from *D*, which means that the untrustworthy intermediate node can be avoided. Ultimately, the trust evaluation chain for node *I* is *D→F→G→I*. This method greatly reduces the length of the trust delivery chain compared with the traditional method, thus improving the credibility of trust evaluation.

### 5.2. Mobile Edge Computing for Trustworthy Data Collection

Accurate and efficient data collection is the basis of management decisions for the trustworthy system. Therefore, it is necessary for a cloud to manage data trust of the underlying sensor network. On the basis of the trust evaluation of sensor nodes above, the trustworthy factor of nodes is considered in the process of planning the moving path of the mobile edge node. Mobile edge nodes are able to avoid untrustworthy sensor nodes, which is especially important when collecting latency-sensitive data because unnecessary movement latency can be avoided. However, considering the slow speed of the mobile node, path planning should consider the overall trust situation within the set area rather than a single node. The combination of the overall situation and the local trust can maximize efficiency, meet the requirements of data timeliness, and help to improve the trustworthy level of sensor-cloud based on mobile edge computing. The mobile edge node should be considered to move to the high trustworthy area as much as possible within a limited moving distance to meet the need to collect more trustworthy data at once, while the network deployment and application platform are not affected.

### 5.3. Mobile Edge Computing for Data Cleaning

Even if the system can access the trustworthy data source via the mobile node for data collection, it still fails to ensure that the data are completely trustworthy. The system needs to further filter the data in the life cycle of data, due to some unreliable factors in the process of data delivery, such as deliberate interference and destruction via malicious nodes. The abnormal data are detected in real time and dynamically via mobile edge nodes in the process of data collection to reserve the trustworthy data and discard the untrustworthy data directly to avoid uploading to the cloud, which not only eliminates the abnormal data, but also saves energy and bandwidth.

The process of data cleaning can be divided into two steps: (a) The data are analyzed and the untrustworthy data are discarded directly. The data collected via sensor nodes generally have two characteristics; namely, space similarity and time similarity. Space similarity refers to the similarity of data collected via nodes with similar geographical locations, and time similarity refers to the similarity of data collected via the same node at a similar time. The mobile edge node is very suitable to undertake the task of local computing in the mobile edge computing model to obtain the information of the two dimensions of similar space and similar time, thereby diagnosing the abnormal data. The edge layer is used to dynamically maintain a spatiotemporal dataset. The outlier detection algorithm is used for analysis based on this spatiotemporal dataset to detect and identify abnormal and untrustworthy data; (b) The mobile edge node can use its own computing power to make a local pre-decision based on the data collected. The mobile edge node merely needs to submit the decision results to cloud if it can make a pre-decision on the event; otherwise, the data should be submitted to the cloud for comprehensive analysis and judgment. For instance, in the application of forest fire monitoring, if the mobile edge node can find the occurrence of fire according to the local monitoring information, then it is not necessary to transmit the data to the cloud for further judgment; only the fire information needs to be reported directly. In the subsequent study, it will be vital to mine deeply the computing power and storage capacity of edge computing combined with sensor-clouds, and utilize the advantage of the edge near to the local to design.

## 6. Conclusions

The rise and development of sensor-clouds expanded the market space of cloud computing and presented excellent application prospects for WSNs. Sensor-clouds also pose new challenges. There are some problems such as low efficiency and difficulty in ensuring data security existing in a sensor-cloud due to network bandwidth and geographic location. Mobile edge computing brings computation resources close to the underlying WSNs, which enables offloading highly demanding computations to mobile edge computing, minimizes energy consumption at WSNs, and reduces the load on clouds. Few studies describe the chances and ideas for the combination of sensor-clouds and mobile edge computing conclusively. Through a more comprehensive investigation and survey, we focused on some challenges of storage, trust evaluation, and data collection in sensor-clouds, which can be mitigated by leveraging mobile edge computing. As another significant contribution of this paper, we identified the main open problems. However, the introduction of new methods will also bring new problems, such as uncertainty and high delay caused by node moving. These new challenges will be the focus of future research.

## Figures and Tables

**Table 1:** Mobile edge-based sensor-cloud.

Research Direction	Research Content	Strategy Method	Contribution	Papers
Architecture	Layered Architecture	LRMC; Event tree based scheduling system	Abatement of events dependencies	[62,63]
Trust	Trust Evaluation	Hierarchical trust mechanism; CTDC; FDS	Optimization of trust evaluation model	[9,31,42,62]
Security	Data Security	TASA; MST based routing method; MMSA	Reduction of latency and energy consumption	[9,44]
	Authentication	Matrix-based key agreement; data encryption	Reduction of redundant authentication and improvement of system efficiency	[64,65,66]
	Transmission Security	CORA; BCRA	Optimization of computing resource allocation	[67,68]
	Coupling Security	Hungarian algorithm	Decrease of coupling computing and increase of resource utilization	[69]
Storage	Hierarchical Storage	Hash-Solomon code algorithm; Reed-Solomon code algorithm	User privacy protection and minimization of communication cost	[67,70]
Service	QoS	FOGPLAN; MINLP	Minimization of the system cost	[71,72]
